# Treatment of human muscle cells with popular dietary supplements increase mitochondrial function and metabolic rate

**DOI:** 10.1186/1743-7075-9-101

**Published:** 2012-11-13

**Authors:** Roger A Vaughan, Randi Garcia-Smith, Miguel A Barberena, Marco Bisoffi, Kristina Trujillo, Carole A Conn

**Affiliations:** 1 Department of Health, Exercise and Sports Science, University of New Mexico, University Blvd, Albuquerque, NM, 87131, USA; 2 Department of Biochemistry and Molecular Biology, University of New Mexico Health Sciences Center, Albuquerque, NM, 87131, USA; 3 Department of IFCE: Nutrition, University of New Mexico, Albuquerque, NM, 87131, USA

**Keywords:** Mitochondrial biosynthesis, PGC, Extracellular acidification and oxygen consumption rates, Metabolic syndrome, Oxidative phosphorylation, Dietary supplements

## Abstract

**Background:**

Obesity is a common pathology with increasing incidence, and is associated with increased mortality and healthcare costs. Several treatment options for obesity are currently available ranging from behavioral modifications to pharmaceutical agents. Many popular dietary supplements claim to enhance weight loss by acting as metabolic stimulators, however direct tests of their effect on metabolism have not been performed.

**Purpose:**

This work identified the effects popular dietary supplements on metabolic rate and mitochondrial biosynthesis in human skeletal muscle cells.

**Methods:**

Human rhabdomyosarcoma cells were treated with popular dietary supplements at varied doses for 24 hours. Peroxisome proliferator-activated receptor coactivator 1 alpha (PGC-1α), an important stimulator of mitochondrial biosynthesis, was quantified using quantitative reverse transcriptase polymerase chain reaction (qRT-PCR). Mitochondrial content was measured using flow cytometry confirmed with confocal microscopy. Glycolytic metabolism was quantified by measuring extracellular acidification rate (ECAR) and oxidative metabolism was quantified by measuring oxygen consumption rate (OCR). Total relative metabolism was quantified using WST-1 end point assay.

**Results:**

Treatment of human rhabdomyosarcoma cells with dietary supplements OxyElite Pro (OEP) or Cellucore HD (CHD) induced PGC-1α leading to significantly increased mitochondrial content. Glycolytic and oxidative capacities were also significantly increased following treatment with OEP or CHD.

**Conclusion:**

This is the first work to identify metabolic adaptations in muscle cells following treatment with popular dietary supplements including enhanced mitochondrial biosynthesis, and glycolytic, oxidative and total metabolism.

## Background

Obesity is an increasingly prevalent morbidity with nearly two thirds of adult Americans overweight, half of whom are obese [1]. Obesity-related health issues have been reported to increase healthcare costs by an estimated $147 billion annually [2]. Over the past decade, chemical and behavioral interventions that favorably modify metabolic rate have been central to obesity research.

Several over-the-counter dietary supplements claim to increase metabolic rate and enhance fatty acid catabolism. Of the available over-the-counter (OTC) dietary supplements, OxyElite Pro (OEP) produced by USP Laboratory and Cellucore Super HD (CHD) are purported to increase metabolic rate and fat metabolism [3].

OEP has been shown to increase markers of fat mobilization, metabolic rate (measured via indirect calorimetry), and reduce bodyweight and body fat (estimated via Dual-energy X-ray absorptiometry) in healthy young subjects following ingestion [3,4]. A key ingredient 1,3-dimethylamylamine (also known as germanium, geranamine or DMAA) has been implicated for potential contraindications. DMAA is purported to increase both systolic and diastolic blood pressure in young and healthy men and women immediately following ingestion, although these observations have been inconsistent during longer treatments with OEP in young men [4-7]. DMAA is also purported to cause false-positive results for amphetamines on select immunoassays, a profound implication for athletes with sanctioned governing bodies [8]. CHD, a newer dietary supplement has very limited research regarding safety or efficacy. CHD is advertised to increase metabolic rate and decrease fatty acid synthesis. Many of the ingredients including caffeine have been previously linked to increased metabolic rate. Moreover, because supplements commonly contain a variety of ingredients in proprietary blend forms, and few controlled studies have been performed to address the metabolic effects at the cellular level, further work is needed to identify possible metabolic effects. This work specifically addresses the effects that treatment with OEP or CHD supplements have on metabolism in human skeletal muscle cells.

Peroxisome proliferator-activated receptor coactivator 1 alpha (PGC-1α) is a transcriptional coactivator that is essential for mitochondrial biosynthesis and activates genes that regulate energy homeostasis and metabolism [9-11]. PGC-1α increases fatty acid oxidation through increased peroxisome proliferator-activated receptor alpha (PPARα) expression, which increases forkhead box protein 1 (FOXO1), nuclear respiratory factors 1 and 2 (NRF1/2) and other factors influencing fat oxidation [12-14]. PGC-1α is also an important signaling molecule in the activation and regulation of gluconeogenesis, which is likely mediated through FOXO1 and estrogen-related receptor alpha (ERR-α) [12,15-18]. Thus, PGC-1α modifies metabolic rate and expression of genes involved in gluconeogenesis, fat oxidation and mitochondrial biosynthesis [12-18].

Clinically, the relationship between low PGC-1α expression and type II diabetes/obesity has been identified [19-22]. Low PGC-1α is also associated with reduced expression of oxidative phosphorylation genes, decreasing fatty acid oxidation and energy utilization [19,23,24]. Treatment with the PGC-1α stimulator Rosiglitazone (through binding and activating PPARγ) increased mitochondrial density and function, while improving insulin sensitivity [25]. Further evidence suggests that an increase in PGC-1α (independent of Rosiglitazone) can improve insulin sensitivity and improve muscle function [25]. It has also been identified that PGC-1α is essential for the recovery from the diminished ATP caused by chemical uncoupling as evidenced by the lack of recovery in PGC-1α null cells and animals [26].

Treatment with potent research-grade chemicals such as 2,4-dinitrophenol (DNP) and p-trifluromethoxy phenylhydrazone (FCCP) have been shown to induce PGC-1α in fibroblasts [26]. Moreover, our laboratory recently identified that treatment with DNP or caffeine can induce PGC-1α, and increase both metabolic rate and mitochondrial content in muscle cells suggesting that commercially available metabolic stimulators might have similar effects [27]. The well documented effects of PGC-1α on metabolism suggest that modulation of PGC-1α expression is a potential strategy for altering metabolic rate.

Purpose- This work seeks to explore effects of treatment with OTC dietary supplements on mitochondrial and glycolytic metabolism in skeletal muscle cells. Human rhabdomyosarcoma cells are a naturally immortalized cell model, frequently used for making inferences about muscle tissue adaptations [27-31]. We show that treatment of muscle cells with OEP or CHD at varied doses induce PGC-1α mRNA and protein in a dose and time sensitive manner. We also illustrate that treatment with either OEP or CHD increase mitochondrial content. This work identifies for the first time the effects that several OTC diet supplements have on mitochondria content and cell metabolism in muscle cells.

## Methods

### Cell culture

*Homo sapiens* rhabdomyosarcoma cells were purchased from ATCC (Manassas, VA). Cells were cultured in Dulbecco’s Modified Eagle’s Medium (DMEM) containing 4500mg/L glucose and supplemented with 10% heat-inactivated fetal bovine serum (FBS) and 100U/mL penicillin/streptomycin, in a humidified 5% CO_2_ atmosphere at 37°C. Trypsin-EDTA at 0.25% was used to detach the cells for splitting and re-culturing. Stock Oxy Elite Pro^TM^ (OEP) from USP Labs (Dallas, TX) and stock Cellucore HD^TM^ (CHD) from Cellucore (Bryan, TX) purchased over the counter were diluted to 2 dilutions that contain equivalent ingredient by weight; high dose containing 90 μg/ml or low dose containing 45 μg/ml. Dose and exposure times were determined through pilot experiments to significantly increase PGC-1α (data not shown). Final concentration of ethanol was 0.1% for all treatments.

### Quantitative real-time polymerase chain reactions (qRT-PCR)

Cells were seeded in 6-well plates at a density of 1 x 10^6^ cells/well, treated and incubated as described above for 12 or 24 hours. Following incubation, total RNA was extracted using RNeasy Kit from Qiagen (Valencia, CA) and total RNA was quantified by Nanodrop spectrophotometry. RNA (5000 ng/sample) was denatured at 75°C for 3 minutes and cDNA was synthesized using random decamers and Moloney murine leukemia virus reverse transcriptase (MMLVRT) from the Retroscript™ RT kit from Ambion (Austin, TX) for 60 minutes at 42°C and the enzyme denatured at 92°C for 10 minutes. PCR primers were designed using Primer Express software from Invitrogen (Carlsbad, CA) and synthesized by Integrated DNA Technologies (IDT; Coralville, IA). For PGC-1α, the forward primer was 5’-ACCAAACCCACAGAGAACAG-3’ and the reverse primer was 5’-GGGTCAGAGGAAGAGATAAAGTTG-3’. Amplification of PGC-1α was normalized to the housekeeping gene, TATA Binding Protein (TBP). For TBP, the forward primer was 5’-CACGAACCACGGCACTGATT-3’ and the reverse primer was 5’-TTTTCTTGCTGCCAGTCTGGAC-3’. qRT-PCR reactions were performed in triplicate using the LightCycler 480 real-time PCR system from Roche Applied Science, (Indianapolis, IN). SYBR Green based PCR was performed in triplicate using an estimated 800 ng of cDNA per well to ensure a strong signal; final primer concentrations were 10 μM in a total volume of 30μl. The following cycling parameters were used: 95°C for 10 minutes followed by 45 cycles of 95°C for 15 seconds, and 60°C for one minute. Relative expression levels were determined by the change in crossing points of reaction amplification (ΔΔCp method) between PGC-1α and TBP for each treatment compared with the control group [32].

### Flow cytometry

Cells were plated in 6-well plates at a density of 1.2 x 10^6^ cells/well treated in triplicate and incubated as previously described above for 24 hours. Following treatment, the media was removed and the cells were re-suspended in pre-warmed media with 200 nM Mitotracker Green from Life Technologies (Carlsbad, CA) and incubated for 45 minutes in a humidified 5% CO_2_ atmosphere at 37°C. The cells were pelleted, the media with Mitotracker was removed and the cells were suspended in pre-warmed media. Group mean fluorescence was measured using Facscalibur filtering 488nm.

### Microscopy and immunohistochemistry

Chamber-slides from BD Bioscience (Sparks, MD), were seeded with 5000 cells/well. To verify PGC-1α protein expression, cells were cultured and treated for 24 hours as described above. Cells were fixed using 3.7% formaldehyde in media, permiabilized with PBS with 0.1% Triton 100X from Sigma (St. Louis, MO) for 10 minutes and blocked for 1 hour with PBS with 0.1% Triton 100X and 3.0% BSA from Sigma (St. Louis, MO). Cells were stained with an anti-PGC-1α primary polyclonal antibody from Santa Cruz Biotechnologies (Santa Cruz, CA) at 1:200 dilution in PBS with 0.1% BSA overnight. The cells were rinsed with PBS with 0.1% Triton 100X and 3.0% BSA, and secondary anti-rabbit AlexFluor 633 antibody from Invitrogen (Carlsbad, CA) was applied in 1:200 dilution. Slides were mounted with Prolong Gold with DAPI from Invitrogen (Carlsbad, CA) and cured overnight. Cells were imaged using the Axiovert 25 microscope with AxioCam MRc from Zeiss (Thornwood, NY). To verify increased mitochondrial content, the cells were then stained with Mitotracker 200 nM from Invitrogen (Carlsbad, CA) for 45 minutes, and fixed in 3.7% formaldehyde in pre-warmed media. Cells were mounted, cured and imaged as described above.

### Metabolic assay

Cells were seeded overnight in 24-well culture plate from SeaHorse Bioscience (Billerica, MA) at density 5 x 10^5^ cells/well, treated and incubated for 24 hours as described above. Following treatment, culture media was removed and replaced with XF Assay Media from SeaHorse Bioscience (Billerica, MA) containing 4500mg/L glucose free of CO_2_ and incubated at 37°C. Per manufactures’ protocol, SeaHorse injection ports were loaded with oligomycin, an inhibitor of ATP synthase which induces maximal glycolytic metabolism and reveals endogenous proton leak (mitochondrial uncoupling) at a final concentration 1.0 μM. Oligomycin addition was followed by the addition of carbonyl cyanide *p*-[trifluoromethoxy]-phenyl-hydrazone (FCCP), an uncoupler of electron transport that induces peak oxygen consumption (an indirect indicator of peak oxidative metabolism) at final concentration 1.25 μM. Rotenone was then added in 1.0 μM final concentration to reveal non-mitochondrial respiration and end the metabolic reactions [33,34]. Extracellular acidification, an indirect measure of glycolytic capacity, and oxygen consumption, a measure of oxidative metabolism was measured using the SeaHorse XF24 Extracellular Analyzer from SeaHorse Bioscience (Billerica, MA). SeaHorse XF24 Extracellular Analyzer was run using 8 minute cyclic protocol commands (mix for 3 minutes, let stand 2 minutes, and measure for 3 minutes) in triplicate.

### WST1 Metabolic assay

WST-1 is a widely used reagent that is metabolized by mitochondrial dehydrogenases through consumption of reduction potential resulting in the fluorescence indicating changes in metabolism, cell proliferation and viability. We used the WST-1 assay as an indirect measure of cellular reduction potential which indicates cellular energy status. Cells were seeded in 96-well plates at density 5,000 cells/well and grown over night. Cells were then treated with either ethanol control, or one of the designated OTC supplement treatments and incubated as previously described above for 24 hours. Media and treatment were removed at each time point and media containing 10% WST1 reagent was added to each well and incubated as previously described above. Fluorescence was measured 1 hour following WST1 addition using Wallac Victor3V 1420 Multilabel Counter from PerkinElmer (Waltham, MA).

### Cell viability

Cells were seeded for 24 hours in 6-well plate with density 1.2 x 10^6^ cells/well and treated in triplicate and incubated as previously described above for 24 hours. Trypan blue from Sigma (St Louis, MO) exclusion staining was used to assess cell number and viability using Countess from Invitrogen (Carlsbad, CA) cell quantification system.

### Statistical analysis

PGC-1α expression, flow cytometry, metabolic assays and cell viability were analyzed using ANOVA with Dunnett’s post hoc test and pairwise comparisons were used to compare treatments with control. WST1 cell metabolism analysis was performed using ANOVA and pairwise comparisons comparing treatments with control following reciprocal transformation of group log fluorescence and data normalization to control (control metabolic rate = 1). Values of *p* < 0.05 indicated statistical significance in all tests and Prism from GraphPad (La Jolla, CA) was used to perform all statistical analyses.

## Results and discussion

### PGC-1α expression and mitochondrial content

**Figure 1:**
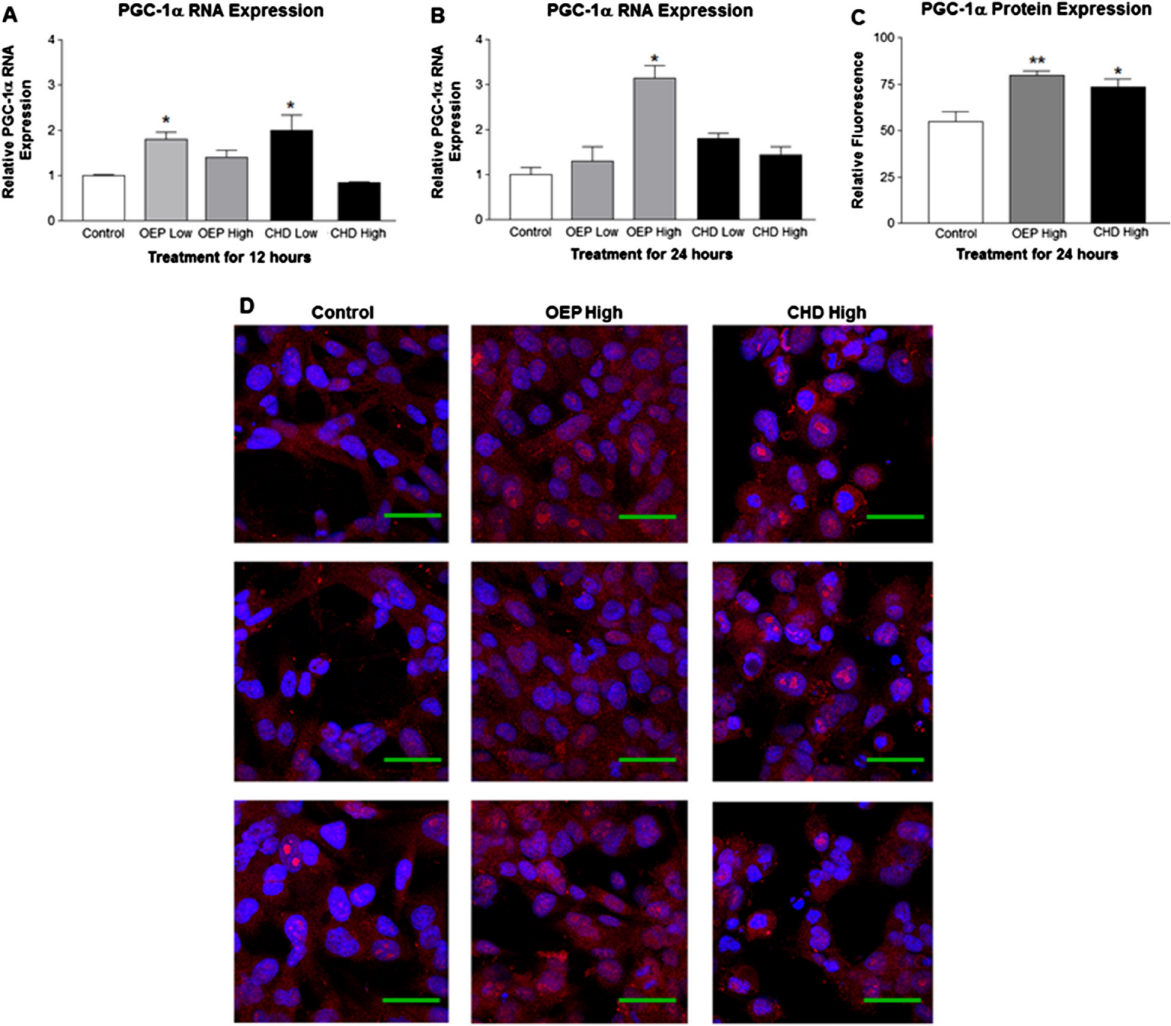
**Group mean PGC-1α RNA and protein expression ±SD.A**- Relative induction of PGC-1α RNA following treatment of rhabdomyosarcoma cells with OEP or CHD at 90 μg/ml (High) or 45 μg/ml (Low) for 12 hours compared with ethanol control (control =1). **B**- Relative induction of PGC-1α RNA following similar treatment for 24 hours. **C**- Immunofluorescent quantification of PGC-1α protein expression from confocal microscopy in (D) using 7 cells per treatment (n = 7) following treatment with OEP or CHD at 90 μg/ml (High) for 24 hours compared with ethanol control. **D**- Confocal microscopy of PGC-1α protein expression of cells treated as described above for 24 hours. NOTES: Dapi nuclear stain (blue), PGC-1α protein (red), and green line represents 50 μm. * indicates *p* < 0.05, ** indicates *p* < 0.01 and *** indicates *p* < 0.001.

**Figure 2:**
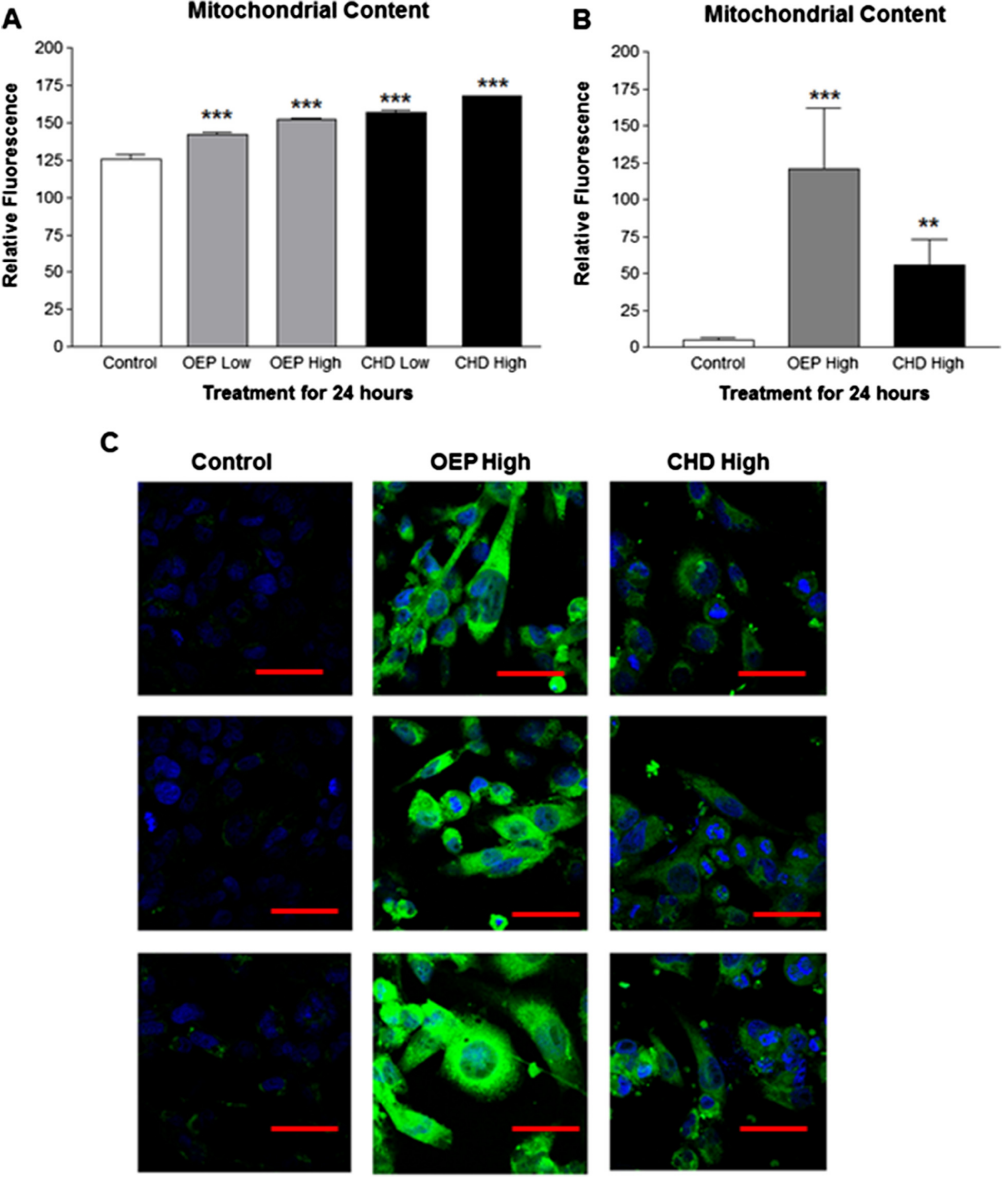
**Group mean mitochondrial content ±SD.A**- Flow cytometric group mean log fluorescence of rhabdomyosarcoma cells measured in triplicate using 10,000 cells per sample, treated with OEP or CHD at 90 μg/ml (High) or 45 μg/ml (Low). **B**- Immunofluorescent quantification of cells treated as described above for 24 hours using 7 cells per treatment (n = 7). **C**- Confocal microscopy images of cells treated as described above for 24 hours. NOTES: Dapi nuclear stain (blue), mitochondrial stain (green), and red line represents 50 μm. * indicates *p* < 0.05, ** indicates *p* < 0.01 and *** indicates *p* < 0.001.

PGC-1α RNA was significantly induced following treatment for 12 or 24 hours with OEPHigh and Low or CHDLow compared with the control group (Figure 1A and B, respectively). PGC-1α protein was imaged using confocal microscopy and quantified with ImageJ using 7 cells per treatment (n = 7) which revealed significantly elevated PGC-1α protein following treatment for 24 hours with OEPHigh or CHDHigh (Figure 1C and D). Flow cytometry with Mitotracker staining showed that treatment for 24 hours with either OEP or CHD significantly increased mitochondrial content compared with ethanol control (Figure 2A). We used confocal microscopy to verify flow cytometry observations that OEP or CHD treated cells had increased mitochondrial content (Figure 1B and C).

### Glycolytic metabolism

**Figure 3:**
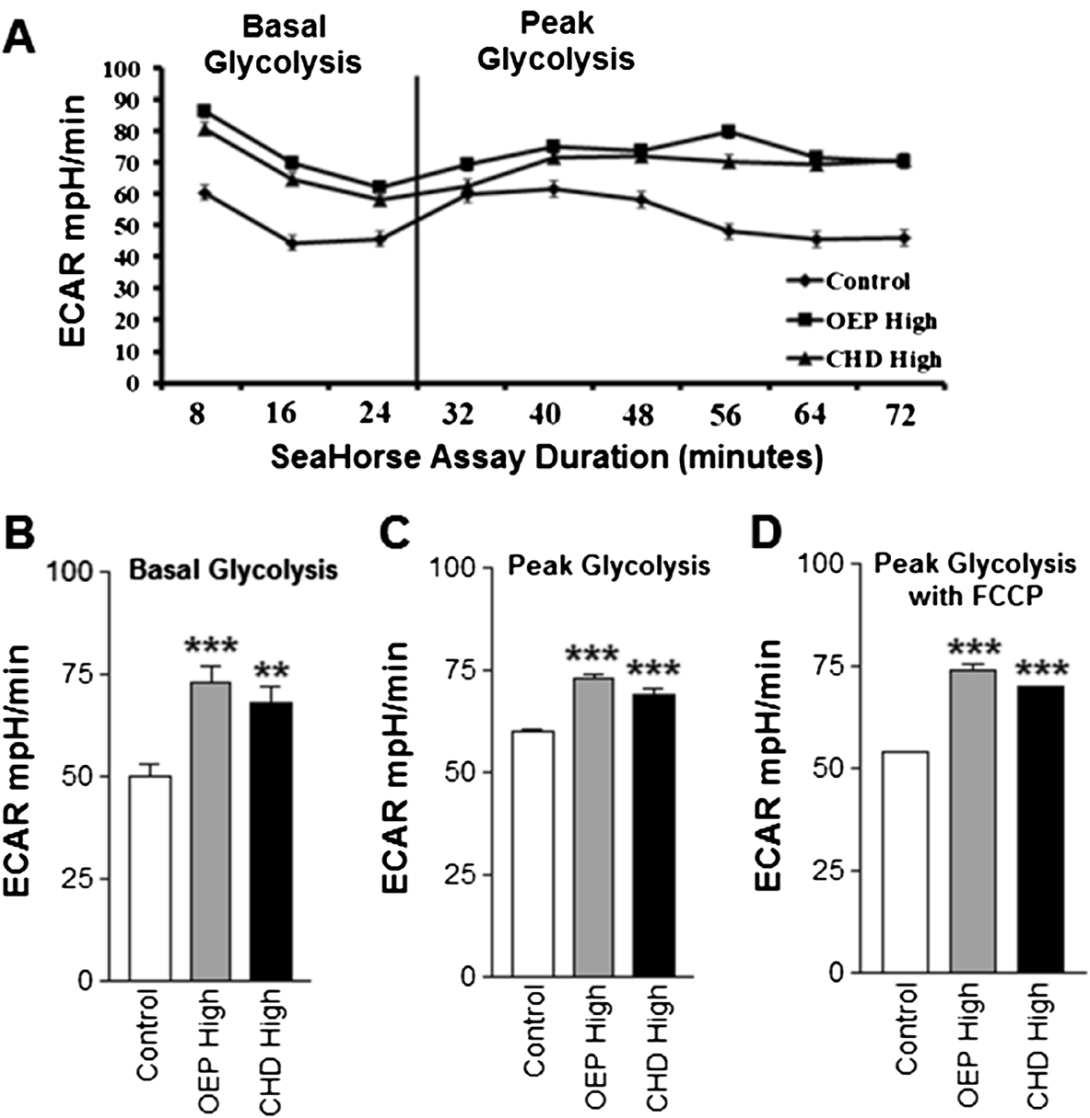
**Group mean glycolytic metabolism ±SD.A**- Extracellular acidification rate (ECAR) of rhabdomyosarcoma cells treated with either ethanol control (final concentration = 0.1%) or OEP or CHD at 90 μg/ml (High) for 24 hours. **B**- Baseline ECAR following treatment described above. **C**- Peak ECAR following addition of oligomycin, an inhibitor of oxidative phosphorylation. **D**- Peak ECAR during peak oxygen consumption rate (OCR) following addition of carbonyl cyanide p-[trifluoromethoxy]-phenyl-hydrazone (FCCP), a mitochondrial uncoupling agent, in addition to previously added oligomycin. NOTES: * indicates *p* < 0.05, ** indicates *p* < 0.01, and *** indicates *p* < 0.001 compared with control.

In order to quantify changes in glycolytic metabolism, we measured extracellular acidification rate (ECAR), which was significantly increased in cells treated with high dose supplements (Figure 3A). Treatment with either OEPHigh or CHDHigh significantly increased basal glycolysis compared with control (Figure 3B). Treatment with either OEPHigh or CHDHigh also significantly increased peak glycolytic capacity compared with control (Figure 3C). OEPHigh or CHDHigh treatment also significantly elevated glycolytic use during induction of peak oxidative metabolism compared with the control group (Figure 3D). Cells treated with low-dose OEP or CHD did not have greater basal or peak glycolytic capacity compared with control (data not shown).

### Oxidative metabolism

**Figure 4:**
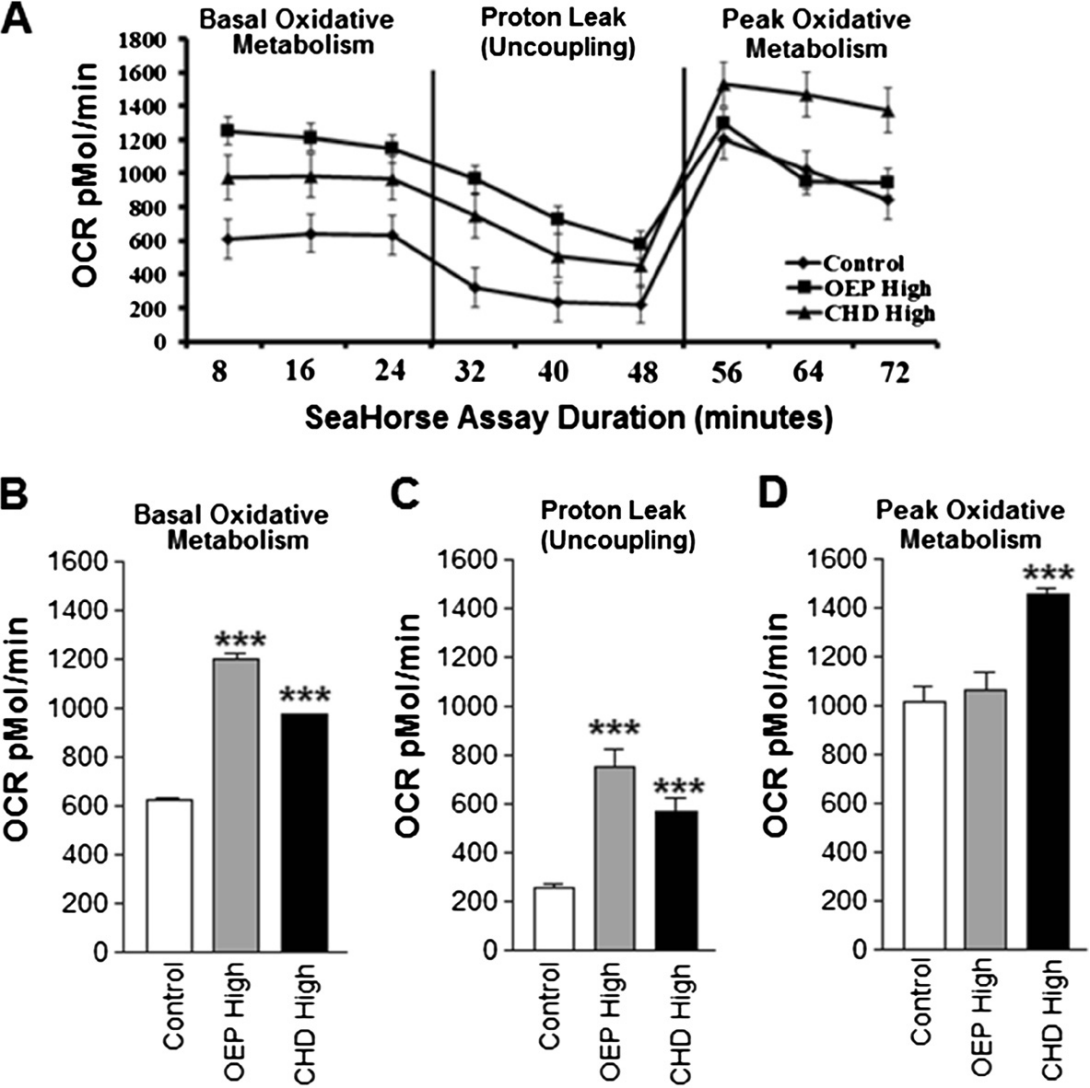
**Group mean oxidative metabolism ±SD.A**- Oxygen consumption rate (OCR) of rhabdomyosarcoma cells treated with either ethanol control (final concentration = 0.1%) or OEP or CHD at 90 μg/ml (High). **B**- Baseline OCR following treatment described above. **C**- OCR during peak ECAR following addition of oligomycin, an inhibitor of oxidative phosphorylation. **D**- Peak OCR following addition of carbonyl cyanide p-[trifluoromethoxy]-phenyl-hydrazone (FCCP), a mitochondrial uncoupling agent, in addition to previously added oligomycin. NOTES: * indicates *p* < 0.05, ** indicates *p* < 0.01, and *** indicates *p* < 0.001 compared with control.

In order to quantify changes in oxidative metabolism, we measured oxygen consumption rate (OCR) which was also elevated in cells treated with high dose OEP or CHD (Figure 4A). Treatment with either OEPHigh or CHDHigh significantly increased basal oxidative metabolism compared with control (Figure 4B). Treatment with either OEPHigh or CHDHigh also significantly increased oxidative metabolism during peak glycolytic capacity compared with control, an indicator of increased endogenous mitochondrial uncoupling (or potentially chemically-induced uncoupling) (Figure 4C). Unexpectedly, only treatment with CHDHigh significantly elevated peak oxidative metabolism compared with the control group (Figure 4D). Cells treated with low-dose OEP or CHD did not consistently exhibit greater basal or peak oxidative metabolism capacity compared with control (data not shown). Only OEPHigh exhibited significantly greater oxygen consumption compared with the control following addition of rotenone, an indirect measure of non-mitochondrial respiration (data not shown).

### Cellular oxidative reliance

**Figure 5:**
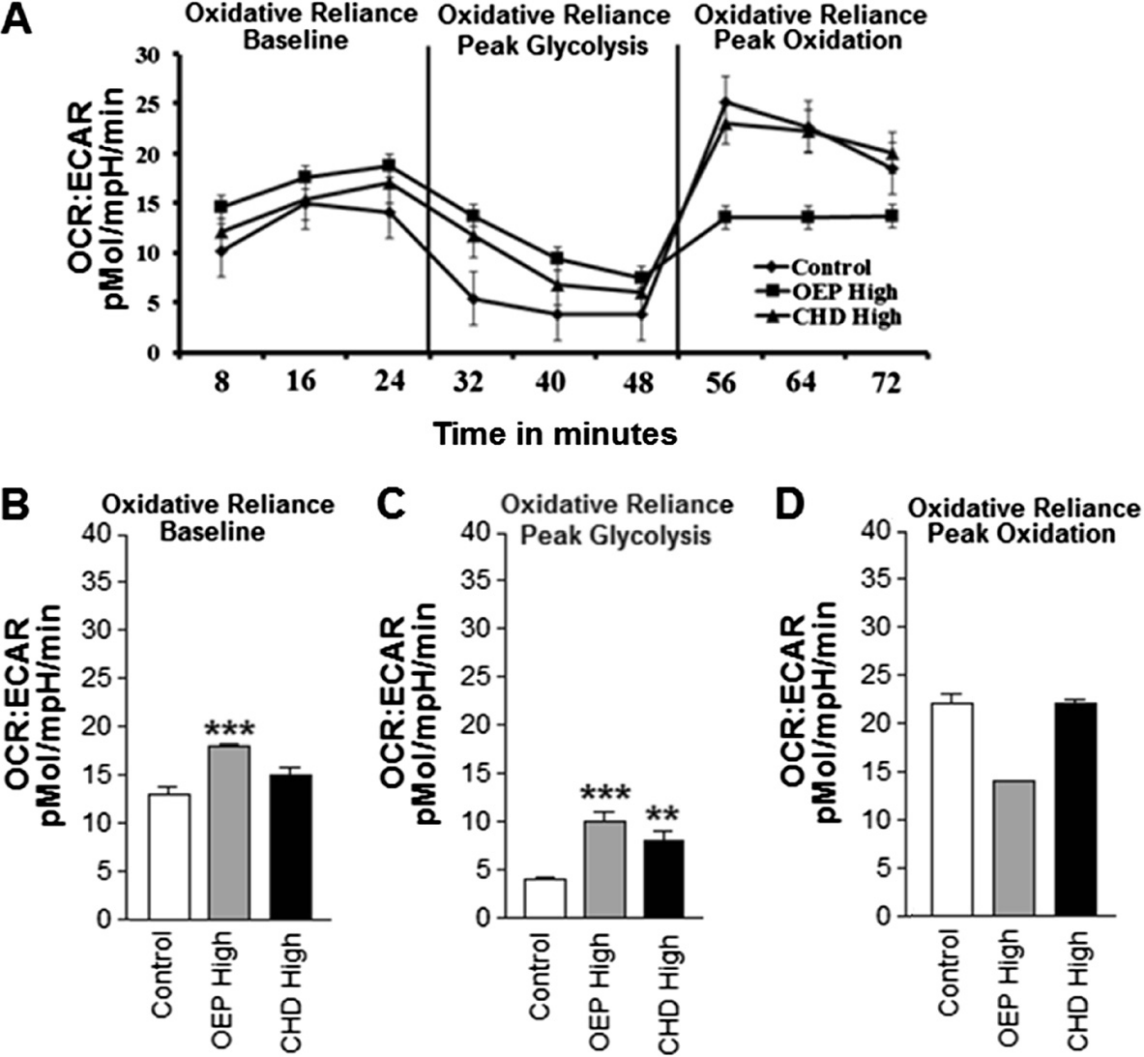
**Group mean oxidative reliance ±SD.A**- Metabolic reliance expressed as the ratio of oxygen consumption rate (OCR) extracellular acidification rate (ECAR) OCR:ECAR illustrating metabolic rate. **B** Oxidative reliance represented by a ratio of OCR:ECAR at baseline. **C**- Oxidative reliance during peak glycolysis. **D**- Oxidative reliance during peak oxidation. NOTES: * indicates *p* < 0.05, ** indicates *p* < 0.01, and *** indicates *p* < 0.001 compared with control.

To quantify changes in cellular reliance on oxidative metabolism, we compared ECAR versus OCR. Oxidative reliance, indicated by a ratio of OCR:ECAR was significantly increased in cells treated with high dose OEP or CHD (Figure 5A). Specifically, cells treated with OEPHigh or CHDHigh demonstrated significantly greater reliance on oxidative metabolism during basal measurements and during peak glycolysis compared with the control group (Figure 5C and D, respectively).

### Basal metabolic rate

**Figure 6:**
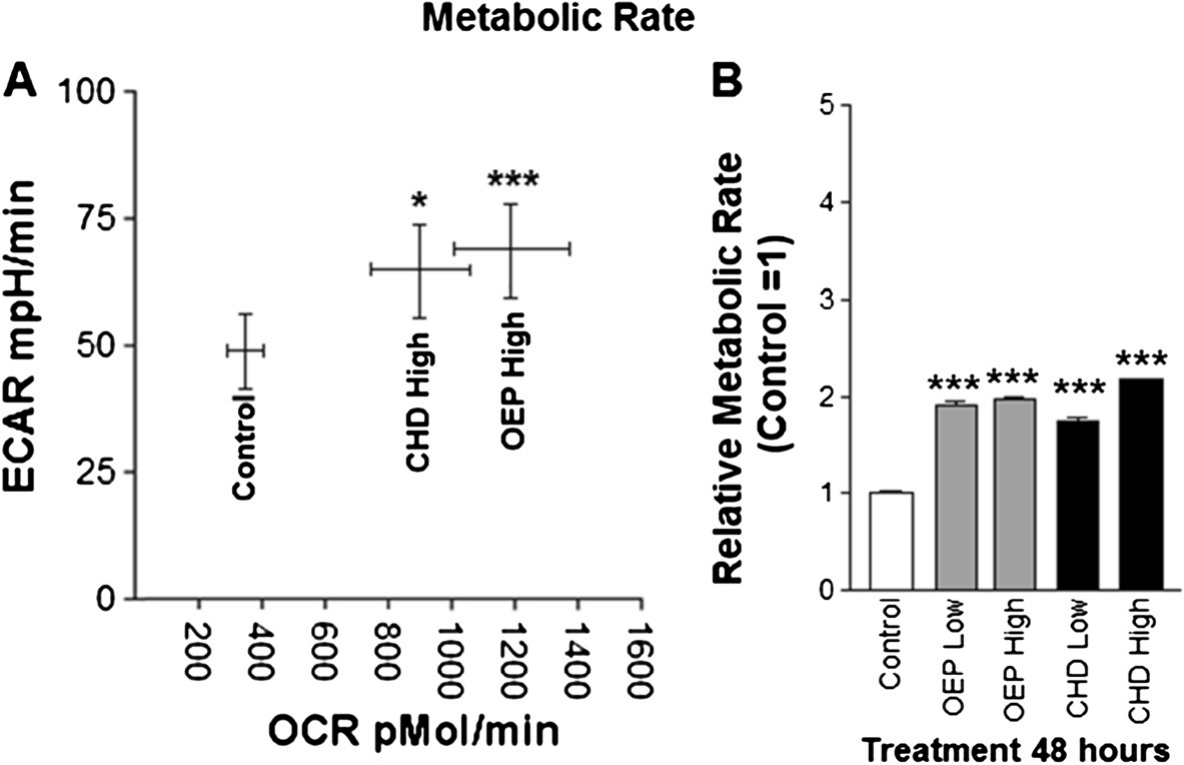
**A- Group mean basal metabolic rate ± SD of rhabdomyosarcoma cells treated with either ethanol control (final concentration = 0.1%) or OEP or CHD at 90 μg/ml (High) plotted as ECAR versus OCR (data from SeaHorse assay).B**- Change in metabolic rate derived from reciprocal log fluorescence from WST1 metabolic assay with control = 1 indicating change in reduction potential within each well treated with either 0.1% ethanol or OEP or CHD at 90 μg/ml (High) or 45 μg/ml (Low). NOTES: * indicates *p* < 0.05, ** indicates *p* < 0.01, and *** indicates *p* < 0.001 compared with control.

To quantify changes in metabolic rate, we compared ECAR versus OCR. Cells treated with OEPHigh or CHDHigh exhibited a significantly greater basal ECAR and OCR, indicating increased metabolic rate (Figure 6A). Moreover, cellular metabolism indicated by WST-1 assay was significantly increased in cells treated with either dietary supplement compared with control. Both OEP and CHD increased metabolism in a dose-dependent manner compared with ethanol control (Figure 6B). To assess the effect of supplement treatment on cell viability, we used Trypan blue exclusion measured by Countess Cell Counter. Following 24 hour of treatment with either ethanol or either of the supplements at either dose, cell viability was not statistically different from control (data not shown).

This work identified several effects that dietary supplements OEP and CHD have on metabolism and cellular adaptation in skeletal muscle cells. First, we illustrated that both dietary supplements increase mitochondrial density and content in part through induction of metabolic transcription factor PGC-1α. Flow cytometry and microscopy experiments verified that cells treated with either supplement exhibited significantly greater mitochondrial content versus ethanol control. We also employed metabolic experiments which identified that both oxidative and total metabolism were significantly increased by both supplements compared with control. WST-1 assay observations in combination with insignificant changes in cell viability also support the observation that both treatments significantly increased cellular metabolism.

Collectively, these results support the notion that stimulators of PGC-1α and mitochondrial metabolism may have implications for many different metabolic diseases such as diabetes and obesity [19-21,25]. Previously, our lab has demonstrated that polyunsaturated fatty acids are dietary constituents which stimulate PGC-1α and mitochondrial metabolism in skeletal muscle cells [35]. Our current observations demonstrate the stimulatory effect of dietary supplements and support several of the previous examinations of the effects of OEP on whole body metabolism [3,4]. This report is among the first to demonstrate that the relatively new dietary supplement CHD also increases metabolism. Lastly, this report is among the first to show an increase in mitochondrial content following treatment with OEP and CHD.

### Limitations

Our experiments were performed using a cancerous myoblast model known to favor glycolytic metabolism as opposed to oxidative metabolism, which may make our observations less generalizable to healthy mature skeletal muscle. Additionally, we prepared culture media per ATCC recommendations containing high glucose; an environment previously shown to induce insulin resistance in L6-myotubes [36]. It would be interesting to examine the effects these supplements elicit from mature, differentiated myotubes in various media. Further research is needed to elucidate the depth of cellular effects that these potent metabolic stimulators may have on metabolic diseases such as diabetes.

## Conclusion

Manipulation of mitochondrial function provides many appealing possibilities for the treatment of several diseases including obesity. This work illustrates that, although much of the biochemical framework has been completed, there is still much to learn about the role that PGC-1α can play in the treatment of various disease states. Further research is needed to identify both behavior modifications and chemical agents that can elicit an increase in mitochondria, because of the role that diminished mitochondria play in chronic diseases. It appears that increased PGC-1α expression and activation accompanied by increased mitochondrial biosynthesis, may be a viable treatment option for diseases such as obesity.

## Competing interests

Roger Vaughan: Was previously employed by General Nutrition Center, a retail agency that sells tested supplements. All authors and contributors declare no other competing interests.

## Authors’ contribution

RV: Performed and over saw all experiments, primary author of manuscript, producer of experimental design, and received Research, Project and Travel Grant to support this project. RG: Assisted with laboratory procedures. MAB: Assisted with laboratory procedures. MB: Assisted with experimental design and manuscript production. KT: Financially supported experiment, assisted with experimental design and manuscript production. CAC: Assisted with experimental design and manuscript production. All authors read and approved the final manuscript.
